# Identification of *Streptococcus gallolyticus* in tumor samples of Iranian patients diagnosed with colorectal cancer

**DOI:** 10.1186/s13104-022-06207-9

**Published:** 2022-10-05

**Authors:** Negin Kamali, Amin Talebi Bezmin Abadi, Bezmin Abadi, Farid Rahimi, Mojgan Forootan

**Affiliations:** 1 Department of Bacteriology, Faculty of Medical Sciences, Tarbiat Modares University, Tehran, Iran; 2 Research School of Biology, The Australian National University, Ngunnawal and Ngambri Country, Canberra, ACT Australia; 3 Gastroenterology and Liver Diseases Research Center, Research Institute for Gastroenterology and Liver Diseases, Shahid Beheshti University of Medical Sciences, Tehran, Iran

**Keywords:** Colorectal cancer, Correlation, Iran, PCR, *Streptococcus gallolyticus*

## Abstract

**Objective:**

Clinical outcomes of infection by *S. gallolyticus* have not been investigated extensively. We aimed to determine the prevalence of *S. gallolyticus* in tumor specimens obtained from Iranian patients diagnosed with colorectal cancer. Polymerase chain reaction was used to confirm the presence of *S. gallolyticus* in patients’ tissue samples.

**Results:**

Of 176 patients, 65 were diagnosed with colorectal cancer whereas 111 did not have any colon disease. No correlation was found between age, colonization with *S. gallolyticus*, gender, or risk factors. Overall, 72 (40%) patients carried *S. gallolyticus*; only 29% of the patients without colorectal cancer were positive for *S. gallolyticus*. Diagnosis of colorectal cancer and presence of *S. gallolyticus* significantly correlated (P = 0.006; odds ratio = 1.46; 95% CI = 1.21–3.87). Among the patients with colorectal cancer, 39 (60%) were positive with *S. gallolyticus* (P = 0.006) whereas 33 of 111 (29.7%) control subjects were positive for *S. gallolyticus* (P > 0.05); thus, 70.3% of the control subjects were not infected with *S. gallolyticus*. We found a high prevalence of *S. gallolyticus* among an Iranian cohort of patients with colorectal cancer. Despite previous reports, we report a positive correlation between colorectal cancer and *S. gallolyticus* colonization.

## Introduction

Diverse species make up the microbiome of the human large intestines, which is estimated to contain 10^14^ aerobic and anaerobic bacteria. The intestinal microbiome interacts with the host mainly symbiotically [1, 2] and contributes to several aspects of human health, including nutrient processing and uptake, metabolism, physiology, and immune function. Disturbing the balanced but complex host–microbiome interactions can cause different diseases including inflammatory bowel disease, hepatic steatosis, obesity, diabetes, and colorectal cancer (CRC) [3].

CRC is the fourth most common cancer reported worldwide [4]. Almost one million new cases of CRC with 600,000 casualties are recorded yearly [5]. CRC, like any cancer, is a multifactorial disease. Cancers initiate by convergence of many interacting underlying factors including colonization by certain infectious agents [6]. As described by Reddy et al. in 1975, CRC occurrence is associated with colonization of intestines by certain bacteria [7], though understandably, every putative association may not represent causation. The mechanisms by which bacterial agents may cause CRC are yet to be discovered..

Microorganisms with carcinogenic potential include opportunistic and anaerobic bacteria, some of which are involved in the early stages of CRC; one of these anaerobic bacteria is *Streptococcus gallolyticus*. *S. gallolyticus* is a gram-positive, opportunistic, immobile, round or oval bacterium [8]. Based on its biochemical properties, *S. gallolyticus* has been classified into three biotypes: biotype I, biotype II/1, and biotype II/2. The biotype I can ferment mannitol; this specific biochemical characteristic distinguishes the biotype I from biotype II [9]. Biotype I corresponds to *S. gallolyticus* subspecies *gallolyticus Sgg* [10]. This subspecies is opportunistic and can cause infective endocarditis, bacteremia, sepsis, hepatic disorders, or gastrointestinal cancers [11]. Though *Sgg* normally inhabits the gastrointestinal tract, present findings about *Sgg* suggest that the pathogenic mechanisms evoked by *Sgg* colonization may lead to CRC [10].

Understanding the mechanisms underlying CRC development requires sophisticated studies. Many studies, including recent meta-analyses have shown that *Sgg* is more common in patients with CRC than in healthy subjects although contradictory studies also abound [12–16]. Collective in vitro knowledge implicates *Sgg* in the CRC development because they are thought to enhance the proliferation of cancer cells through the β-catenin signaling pathway as studied in the murine colon cells [6]. *Sgg* may cause disease also because it highly depends on the type IV collagen in the colonic mucosa and stimulates expression of the proinflammatory cytokines (for example, interleukin-1, interleukin-8, and cyclooxygenase-2 via NF-κB activation) that enhance cell proliferation and angiogenesis by inhibiting apoptosis, thus leading to the CRC development [11].

In this cross-sectional, descriptive, and analytical study, we aimed to investigate the prevalence of *Sgg* among the CRC tissue samples obtained from Iranian patients.

## Main text

### Patients and methods

We enrolled 176 patients who were referred to the Colonoscopy Unit of the Mehrad Hospital, Tehran, Iran, from June 2019 to August 2020. The patients underwent colonoscopy after clinical examination by a physician and completed the informed consent forms and a questionnaire on demographic characteristics and history of drug prescriptions. A skilled gastroenterologist diagnosed the cases based on the patients’ clinical presentations and pathological examinations. All patients were admitted for curative interventions. Biopsy samples (n = 352, two specimens per patient) were collected. The first biopsy sample was fixed in 10% buffered formalin for pathological examinations; the second sample was collected in a 2-mL cryotube containing the thioglycollate broth and transported to the microbiology laboratory within 4 h of colonoscopy for DNA extraction and PCR. During surgical sampling, the colonoscope and surgical instruments were frequently disinfected. The study protocols were reviewed and approved by the Ethics Committee of the Tarbiat Modares University, Tehran, Iran (Ethics application: IR.MODARES.REC.1398.232) and complied with the principles of the Declaration of Helsinki. All patients participated voluntarily and were allowed to leave the study if they wanted.

The second sample of each patient was used for extraction of genomic DNA by using a commercial kit (Roje Technologies, Yazd, Iran); extracted DNA was purified according to the manufacturer’s instructions. The concentration of purified DNA was estimated at λ = 260 nm using a spectrophotometer (WPA, Biochrom, UK). DNA was stored in a freezer at − 20 °C until used. DNA extraction and PCR controls were the β-globin sequence as previously described [14]. To design the PCR primers, the DNASTAR Lasergene software (https://www.dnastar.com/software/lasergene/) was used to find a conserved sequence. Different factors like primer melting temperature, a reasonable G + C content, and low probability of primer-dimer formation were analyzed. Primers listed in Table 1 were added in a 15-µL reaction mix containing 1 µL of bacterial chromosomal DNA (≈ 180 ng), 0.5 µM of each primer, 1 mM of each dNTP, 1.5 mM MgCl_2_, and 0.10 U/µL Taq DNA polymerase. The PCR reaction was performed in a T100 thermal cycler (Bio-Rad, Hercules, CA, USA). *RecA* as a conserved genetic region was selected to confirm the *Sgg* colonization in patients’ samples. To ensure the validity of the PCR experiments, a second operator rechecked 20 samples randomly. We used the ATCC 49475 and a clinically characterized *S. gallolyticus* strain as positive controls in the PCR experiments.

**Table 1:** Primers used in the study

Primer	Oligonucleotide sequence (5’–3’)	Tm (°C)	Product (bp)	References
*rec* -F *rec* -R	TGGTCAAGCTCAGCACCAAT TACACAAGCCAGACGGTTCC	67	361	This study
β*-*globin	ACACAACTGTGTTCACTAGC CAACTTCATCCACGTTCACC	50	110	[14]

All PCR products were visualized following electrophoresis on 2% agarose gels (Sina-clon, Tehran, Iran) in TAE buffer (40 mM Tris, 20 mM acetic acid, and 1 mM EDTA, pH 8.0), staining with GelRed, ultraviolet transillumination (Biometra, Germany), and digital imaging.

Statistical analyses, including correlations between the presence of *recA* and CRC diagnosis, were performed using *VassarStats: Website for Statistical Computation* (http://vassarstats.net/odds2x2.html). p < 0.05 was taken as statistical significance. Student’s *t*-test was used to analyze the correlations among different factors examined in the study.

## Results and discussion

Out of 176 enrolled patients, based on colonoscopy and pathology findings, 65 were diagnosed with CRC; 111 had no record of CRC and were included as the control group. The participants were 40–60 years old; 56% and 43% of patients were male and female, respectively. All the patients’ samples were positive for β-globin by PCR. No significant association was observed between family history of cancer, age, and gender (P > 0.05). The prevalence of *Sgg* among the biopsy samples obtained from 176 patients was 40% (72/176). Importantly, of the patients with CRC, 39 were positive for *S. gallolyticus* (60%) (P = 0.006); meanwhile, 33 of the 111 control subjects (29.7%) were positive for *Sgg* (P > 0.05).

*Sgg* is an intestinal inhabitant in herbivores and was initially isolated from Koala (*Phascolarctos cinereus*) feces [10]. *Sgg* colonization in *Homo Sapiens* was documented by several researchers [17, 18], while ultrastructural studies have shown the role of several putative protein products of the pilus operons in facilitating bacterial attachment to the intestinal epithelium [19]. *Sgg* may serve as a promoter for initiation of CRC in infected cases [6, 10, 20]. Undoubtedly, CRC pathogenesis is multifactorial, and diverse mechanisms, including susceptible genetic traits, diet, infectious agents, and other environmental factors are implicated [21, 22]. Epidemiologic studies support the hypothesis that the risk of CRC may be high in patients colonized with *Sgg* [17, 23, 24]. Kumar et al. reported that *Sgg* contributes to the CRC development by promoting proliferation of the cancer cells [6]. Furthermore, direct interactions between the bacteria and intestinal cells may possibly promote proliferation of the cancer cells in subjects colonized with *Sgg* [25]. In this study, we confirmed the presence of *Sgg* in samples obtained from patients with CRC or non-CRC control subjects to better understand the correlation between colonization by this bacterium and CRC diagnosis. We enrolled 176 cases, one of the largest cohorts studied in Iran. Interestingly, we found that 60% of the patients with CRC were infected with *Sgg* (P = 0.006). In contrast, Mahmoudvand et al. reported 0% (0 out of 6) in colorectal adenocarcinoma biopsy tissues [14]. The differences between these studies could be explained by different population genetics or socioeconomic backgrounds of the studied cohorts. In disagreement with our study, a Malaysian group showed that 24% of all patients with CRC were infected with *Sgg* [26]. A quantitative study by Franch et al. showed that only 3.2% of a cohort of patients with CRC carried *S*. *gallolyticus* [4]. Despite geographical differences (Iran versus Spain), the method applied by Franch et al. differed from that in our study; therefore, such differences overshadow our understanding of the ultimate role of *Sgg* in CRC development. In an interesting study from southwest Iran, Sheikh et al. examined *Sgg* colonization in patients with CRC by stool sampling and PCR [27]. They found that (9/66) 13.6% of the patients were positive for *Sgg*. This finding highlights that *Sgg* can successfully colonize the non-CRC patients; however, we detailed comparative analyses are needed to elucidate the microbe–host interactions during the pathogenesis of CRC. In 2020, Eshaghi et al. reported that 5.5% of patients with CRC were positive for *Sgg* by PCR, the same approach was used in our study [28]. Additionally, Eshaghi et al. explored bacterial culturing as a comparison with PCR findings; they concluded that simultaneous use of both methods is not required to confirm this infection among the clinical samples, and a single method is sufficient [28]. Previously, we have encountered difficulties with culturing *Sgg* from both colon biopsies and stool samples. Thus, the late-growing nature of this bacterium encouraged us to exclude the culture method for detecting *Sgg* in samples but rely only on PCR. Our study is novel because we report a high prevalence of *Sgg* by PCR among an Iranian cohort of patients with CRC. Indeed, our understanding of bacterial causes of CRC or its association with occurrence of a specific bacterial colonization may guide the management of CRC. Sophisticated studies using molecular and biochemical analyses of patients’ samples will improve our understanding of the pathogenesis of chronic life-threatening illnesses such as CRC.

### Limitations

Our study has some limitations. We had aimed initially to enroll at least 100 patients with CRC but could not achieve that target number because the COVID-19 pandemic hindered the late stages of our study. Although our descriptive analysis of the clinical samples does not clarify a factual role for *Sgg* in the CRC development, we postulate that this bacterium is associated with CRC development either epidemiologically or causatively. Despite some contradictory findings, proving an unequivocal association between CRC and *Sgg* requires further molecular, biochemical, and mechanistic studies.

## Data Availability

All data generated or analyzed during this study are included with this article.

## List of abbreviations

CRC: Colorectal cancer.
CI: Confidence interval.
OR: Odds ratio.
*S. gallolyticus*: *Streptococcus gallolyticus*.
